# Supplementary figures and images for: LC/MS-Based Untargeted Metabolomics Study in Women with Nonalcoholic Steatohepatitis Associated with Morbid Obesity
Source: Int J Mol Sci. 2023 Jun 6;24(12):9789. doi: 10.3390/ijms24129789 (PMC10298321; doi:10.3390/ijms24129789)

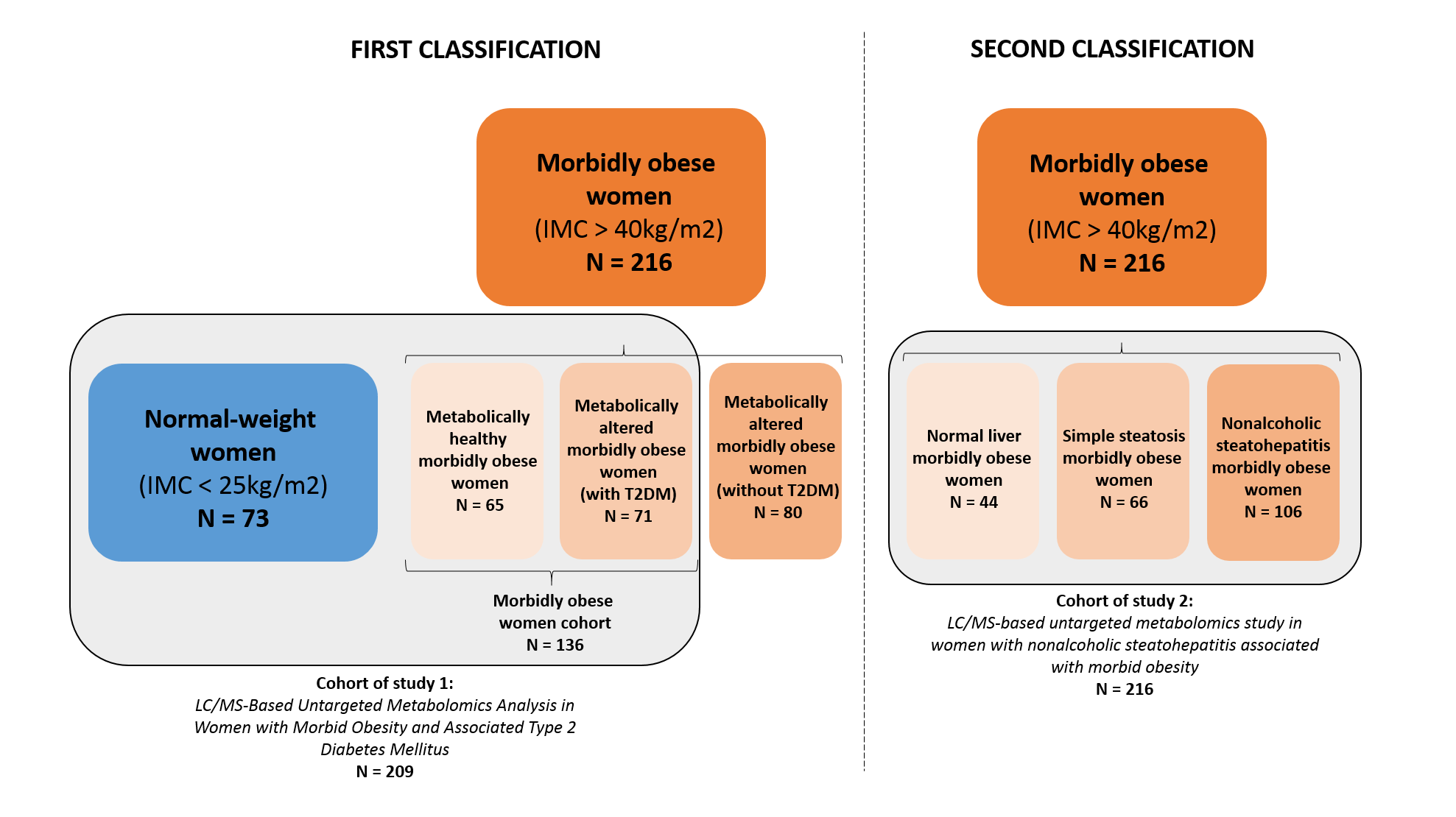

Supplement: Supplementary file 1 [file ijms-24-09789-s001.zip › Figure S1.png]
